# Poster Session I - A68 VALIDATION OF THE SAURIN CLASSIFICATION IN A NORTH AMERICAN PATIENT COHORT UNDERGOING VIDEO CAPSULE AND ENTEROSCOPY FOR OBSCURE BLEEDING

**DOI:** 10.1093/jcag/gwaf042.068

**Published:** 2026-02-13

**Authors:** N Arbabzada, J Reeve, S Wasilenko, K Oguro, S Zepeda Gomez, B Halloran

**Affiliations:** Medicine, University of Alberta, Edmonton, AB, Canada; Medicine, University of Alberta, Edmonton, AB, Canada; Gastroenterology, University of Alberta, Edmonton, AB, Canada; Gastroenterology, University of Alberta, Edmonton, AB, Canada; Medicine, University of Alberta, Edmonton, AB, Canada; Medicine, University of Alberta, Edmonton, AB, Canada

## Abstract

**Background:**

The evaluation of patients with obscure gastrointestinal bleeding (OGIB) frequently involves video capsule endoscopy (VCE) and/or CT enterography (CTE) followed by enteroscopy (including balloon assisted enteroscopy (BAE) and push enteroscopy) for lesion characterization and therapeutic intervention, yet standardized reporting systems are seldom used. The validated Saurin scoring system is used to characterize lesions seen on VCE based on their bleeding potential – P0-P3 with clinically significant lesions scoring P2 or higher. To our knowledge, there is no published literature in its application to North American patient cohorts.

**Aims:**

We aim to assess and validate the Saurin classification in a cohort of patients who have undergone both VCE and BAE. Our objectives are to evaluate the concordance between VCE and enteroscopy for clinically significant lesions (P2 or higher) based on Saurin scoring.

**Methods:**

We performed a retrospective review at a quaternary centre (University of Alberta Hospital) which included adult patients with OGIB who underwent VCE followed by enteroscopy (push or BAE) from January 2010 to May 2024. All procedures were reviewed, and a Saurin score was assigned to both VCE and enteroscopy reports.

**Results:**

A total of 119 patients underwent a VCE followed by enteroscopy with a median age of 65 (range 20-92) and a male predominance (n = 71, 60%) totaling 140 pairings. There were 24 P1, 89 P2 and 25 P3 lesions on VCE accounting for a diagnostic yield of 98.5% (n = 138/140) with 76.3% (n = 87/114) accuracy in predicting clinically significant lesions confirmed by enteroscopy (p < 6.2x10^-7^; OR 10.5) with a false negative rate of 6.5%. The most common diagnosis was vascular lesions (n = 85, 61%). In 18 P2 vascular lesions on VCE (12.9%), the subsequent enteroscopy was unremarkable (7 normal studies and 11 P1 lesions). The pooled diagnostic yield of enteroscopy was 87.1% (n = 122/140) and its therapeutic yield was 55% (n = 77/140). Within the therapeutic subgroup, the yield was considerably higher for P2 and P3 classified lesions compared to those classified as P1 or lower (81% vs 4%, p < 2.2x10^-16^).

**Conclusions:**

The Saurin scoring system is a tool which works well when applied to this North American cohort of patients. If enteroscopy had been performed only in patients with high-risk VCE findings (P2 and P3), we would have spared 26 patients (21.8%) from an invasive procedure while missing only six cases with significant lesions. Validated scoring should become a standardized part of small bowel endoscopy reporting to help improve patient outcomes and resource utilization. Prospective studies are required for further validation.

**Funding Agencies:**

None

